# Investigation of Serum Visfatin and Chemerin Levels in Type 2 Diabetes and Obesity Patients: Their Potential Role as Clinical and Biomarkers

**DOI:** 10.3390/biomedicines13112619

**Published:** 2025-10-26

**Authors:** Duygu Tozcu Yilmaz, Mehmet Ali Gul, Mustafa Capraz, Hatice Dortok Demir, Akin Tekcan

**Affiliations:** 1 Department of Physiology, Faculty of Medicine, Amasya University, 05100 Amasya, Turkey; 2 Department of Medical Biochemistry, Faculty of Medicine, Amasya University, 05100 Amasya, Turkey; mehmetali.gul@amasya.edu.tr (M.A.G.); hatice.demir@amasya.edu.tr (H.D.D.); 3 Department of Internal Diseases, Faculty of Medicine, Amasya University, 05100 Amasya, Turkey; m.capraz@amasya.edu.tr; 4 Department of Medical Biology, Faculty of Medicine, Amasya University, 05100 Amasya, Turkey; akin.tekcan@amasya.edu.tr

**Keywords:** visfatin, chemerin, type 2 diabetes mellitus, obesity, adipokines

## Abstract

**Background/Objectives**: The global prevalence of obesity and type 2 diabetes mellitus (T2DM) has been steadily increasing, and these interrelated disorders share common pathophysiological mechanisms, including altered levels of adipokines secreted from adipose tissue. Among these, chemerin and visfatin have been suggested as potential biomarkers for obesity-related metabolic dysfunction. This study aimed to evaluate the relationship between serum chemerin and visfatin levels and obesity in patients with T2DM. **Methods**: The study included 74 obese T2DM patients, 60 non-obese T2DM patients, and 36 healthy controls. Serum chemerin and visfatin levels were measured using an enzyme-linked immunosorbent assay (ELISA). Clinical parameters including HbA1c, fasting plasma glucose, insulin, and Homeostatic Model Assessment of Insulin Resistance (HOMA-IR) were assessed. Between-group comparisons were performed using appropriate parametric or non-parametric tests with Bonferroni correction for multiple comparisons. ROC curve analysis was applied to evaluate the diagnostic performance of visfatin. **Results**: Serum visfatin levels were significantly higher in the T2DM (33.00 ± 20.61) groups compared to controls (30.25 ± 26.40; *p* = 0.01), while chemerin levels showed no significant difference. HbA1c and glucose levels were elevated in both diabetic groups, whereas insulin and HOMA-IR were significantly higher only in the obese T2DM group. Receiver operating characteristic (ROC) analysis revealed limited diagnostic accuracy of visfatin (AUC < 0.70). **Conclusions**: Visfatin levels were modestly higher in obese T2DM patients, while chemerin did not differ significantly among groups. However, the diagnostic performance of visfatin was limited (AUC < 0.70), and these findings should be regarded as exploratory. Larger, well-controlled studies are required to clarify whether visfatin or chemerin could have any clinical utility as part of multi-marker approaches.

## 1. Introduction

Despite the undeniable improvement in living conditions brought about by modern life, urban and socio-economic development, there have been concomitant problems. These include irregular and unhealthy diets, sedentary lifestyles and increased stress. In the last two decades, obesity has become a global health problem that affects almost all body systems and has reached life-threatening dimensions. Today, obesity is the most common non-communicable public health problem and is a chronic and multifactorial metabolic disease caused by genetic, environmental, psychological and social factors [1,2]. Obesity can cause disturbances in lipid and carbohydrate metabolism, alterations in insulin signaling, pathological enlargement of visceral white adipose tissue (vWAT) and inflammation [3,4,5]. Obesity and type 2 diabetes (T2DM) are closely linked, with obesity being a major risk factor for the development of T2DM [2]. This strong relationship has led to the emergence of the term ‘diabesity’ [6,7]. Despite the wealth of information available on the aetiology of diabetes and obesity, it is evident that certain aspects remain to be elucidated. In recent years, the role of adipose tissue in metabolic and inflammatory diseases, including diabetes, obesity and insulin resistance, has garnered significant attention [8,9]. Adipose tissue is the body’s largest fuel reserve and provides an important portable source of energy that is critical for survival when food is in short supply [10].

Recently, however, adipose tissue has been increasingly recognised as an active endocrine organ. Adipose tissue produces several proteins (adipokines) such as leptin, adiponectin, visfatin, chemerin, and resistin, which have been shown to play an important role in the pathogenesis of insulin resistance, diabetes, obesity, dyslipidaemia, inflammation and atherosclerosis [11], and these adipokines regulate many physiological functions such as energy balance, insulin sensitivity, glucose homeostasis, regulation of food intake, regulation of blood pressure and inflammatory response [12]. Among these, chemerin and visfatin have gained attention for their involvement in obesity-related insulin resistance and inflammatory pathways.

Chemerin is secreted mainly by white adipose tissue, but also by the liver, placenta, and several other organs [13,14,15]. As a novel adipokine, it exerts endocrine, autocrine, and paracrine functions and is implicated in adipogenesis, angiogenesis, obesity, and glucose homeostasis [16,17,18].

Many studies have reported that the levels of chemerin are higher in patients with T2DM compared to healthy individuals, while some studies have reported that there is no significant difference [19,20].

Visfatin, also known as nicotinamide phosphoribosyltransferase (NAMPT) or pre-B cell colony enhancer factor (PBEF), is secreted primarily by adipocytes and macrophages, with lower expression in several other tissues [21,22]. Several clinical studies have investigated its association with insulin resistance, diabetes, and obesity, but results remain inconsistent; some demonstrated a positive correlation with body mass index (BMI) [23,24,25], whereas others did not confirm this relationship [3].

Despite numerous studies investigating visfatin and chemerin separately in metabolic disorders, few have simultaneously evaluated these adipokines in both obese and non-obese patients with T2DM within the same cohort. Given the conflicting evidence regarding their associations with obesity, insulin resistance, and glycemic control, this study aimed to comparatively assess serum visfatin and chemerin levels in these groups and to explore their potential diagnostic relevance.

## 2. Materials and Methods

### 2.1. Study Design and Population

This cross-sectional study was conducted at the Department of Internal Medicine of Amasya University Sabuncuoğlu Şerefeddin Training and Research Hospital between 08/2021 and 08/2022. The study population consisted of 170 participants, categorized into three groups: 74 obese patients with T2DM, 60 non-obese patients with T2DM, and 36 healthy individuals without diabetes or obesity. Inclusion criteria were: male and female patients aged 18–65 years, diagnosed with T2DM, with BMI ≥ 30 kg/m^2^ (obese group) or <30 kg/m^2^ (non-obese group), and healthy controls who voluntarily agreed to participate. Exclusion criteria included: age < 18 or >65, renal/hepatic failure, rheumatologic or inflammatory diseases, thyroid dysfunction, heart failure, chronic infection, pancreatic disorders, use of drugs affecting insulin secretion/sensitivity, pregnancy or lactation, and unwillingness to participate. Control subjects were selected among healthy volunteers without diabetes, cardiovascular, renal, or hepatic diseases. The groups were similar in overall age and sex distribution, although minor variations existed.

The diagnosis of T2DM was made in accordance with the American Diabetes Association (ADA) criteria. The term ‘obesity’ was defined as a BMI of 30 kg/m^2^ or greater. Anthropometric measurements, including weight, height, and waist circumference, were obtained using a body composition analyser (Tanita MC-780MA, Tokyo, Japan) to ensure standardized calculation of body mass index (BMI). Additional outputs provided by the device (e.g., fat mass, fat-free mass, visceral fat) were not systematically recorded across all participants and were therefore excluded from the analysis. Demographic and clinical information including age, sex, medication use, smoking and alcohol habits, diet, and comorbidities were recorded. These lifestyle-related variables were collected for descriptive purposes only and were not used as covariates in statistical analyses, given the cross-sectional design of the study.

### 2.2. Ethical Approval

This study was approved by the Amasya University Non-Interventional Clinical Research Ethics Committee (Date: 3 June 2021, Approval Decision Number: 6/102). Written informed consent was obtained from all participants prior to their inclusion in the study. Furthermore, prior to the initiation of the study, institutional approval regarding the use of patient data was obtained from Amasya University Sabuncuoğlu Şerefeddin Training and Research Hospital (Date: 4 August 2021, Document Number: E-62949364-929).

### 2.3. Sample Collection and Biochemical Analysis

Venous blood samples were collected in yellow-capped serum separator tubes after an 8–12 h fast. The tubes were inverted with caution on five to six occasions, after which the clotting process was permitted to occur at room temperature for a period of 30 min. Thereafter, the tubes were subjected to a centrifugal force of 1500–2000× *g* for a duration of 10 min. The resulting serum was then aliquoted into Eppendorf tubes and stored at −80 °C until biochemical analysis. Serum visfatin and chemerin concentrations were measured using commercially available ELISA kits (Bioassay Technology Laboratory, Birmingham, UK), in accordance with the manufacturer’s protocols. The measurements were performed using a Chromate 4300 ELISA reader (Awareness Technology, Palm City, FL, USA), and all samples were analyzed in duplicate. According to the manufacturer’s information, the detection range for chemerin was 10–3000 ng/L, with a sensitivity of 4.99 ng/L, intra-assay CV < 8%, and inter-assay CV < 10%. For visfatin, the detection range was 0.5–100 ng/mL, with a sensitivity of 0.23 ng/mL, intra-assay CV < 8%, and inter-assay CV < 10%. The mean values were used for statistical analysis.

### 2.4. Statistical Analysis

Statistical analyses were performed using IBM SPSS Statistics 20.0 (IBM Corp., Armonk, NY, USA). For data with normal distribution, one-way ANOVA was used with Bonferroni post hoc tests. For data with non-normal distribution, the Kruskal–Wallis test was used, followed by Bonferroni-corrected Mann–Whitney U tests when significant. Bonferroni correction was applied to all three pairwise comparisons per variable (significance threshold *p* < 0.017). Categorical variables were analyzed using the chi-square test. Since insulin was not measured in the control group for ethical and financial reasons, insulin and HOMA-IR were only compared between obese and non-obese T2DM groups. HbA1c, fasting plasma glucose, insulin, and HOMA-IR results were compared between groups as applicable.

ROC analysis was conducted to evaluate the diagnostic performance of serum visfatin in differentiating obese patients with T2DM from healthy controls. The area under the curve (AUC), sensitivity, and specificity were calculated. AUC values were interpreted as follows: <0.70 = low accuracy, 0.70–0.90 = moderate, and >0.90 = high accuracy.

To provide additional clinical interpretation, effect sizes were calculated for visfatin comparisons using Cohen’s d. Very small effect sizes were observed across all group comparisons, supporting the limited diagnostic value of visfatin. Data are presented as mean ± standard deviation (SD), and *p*-values < 0.05 were considered statistically significant. Data are presented as mean ± standard deviation (SD), and *p*-values < 0.05 were considered statistically significant.

## 3. Results

The study included 74 obese T2DM patients, 60 non-obese T2DM patients, and 36 healthy controls. There were no statistically significant differences between the groups in terms of age, height, smoking status, alcohol consumption, family history of diabetes, or comorbidities. However, sex distribution differed significantly among the groups (Pearson χ^2^ = 15.06, df = 2, *p* = 0.001), primarily due to the markedly higher proportion of men in the non-obese T2DM group (68.3%), whereas the other groups showed a more balanced or slightly male-dominant distribution (Table 1).

Obese T2DM patients had significantly higher values in anthropometric measures compared to both the control and non-obese T2DM groups. Specifically, weight (89.71 ± 14.63 kg), BMI (35.96 ± 5.69 kg/m^2^), and waist circumference (105.60 ± 9.44 cm) were significantly elevated in the obese T2DM group (*p* < 0.001 for all comparisons) (Table 1).

Serum visfatin levels were significantly higher in obese T2DM patients (33.00 ± 20.61 ng/mL) compared to healthy controls (30.25 ± 26.40 ng/mL; *p* = 0.01). No statistically significant difference in visfatin levels was observed between non-obese T2DM patients and the control group or between obese and non-obese patients. Effect size analyses revealed very small differences in visfatin levels across the groups (Cohen’s d = −0.16 for Control vs. Non-Obese T2DM; −0.12 for Control vs. Obese T2DM; +0.06 for Non-Obese vs. Obese T2DM), indicating limited clinical relevance despite statistical significance, and non-parametric tests were used to minimize the influence of potential outliers. Serum chemerin levels did not differ significantly among the groups (*p* = 0.42), although slightly lower values in obese T2DM patients were noted (Table 1). Effect size analyses confirmed that differences in chemerin levels between groups were negligible (Cohen’s d = 0.06 for Control vs. Non-Obese T2DM; 0.14 for Control vs. Obese T2DM; 0.07 for Non-Obese vs. Obese T2DM).

### 3.1. ROC Analysis

**Figure 1:**
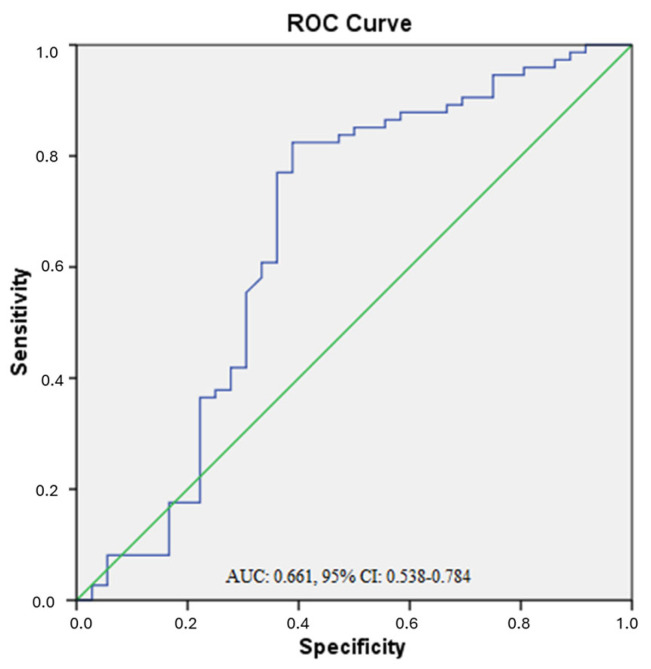
ROC curve of serum visfatin for discriminating obese T2DM patients from controls. ROC curve showing the diagnostic performance of the model (blue line). The diagonal green line represents the reference line (random classifier).

ROC analysis was performed to evaluate the diagnostic accuracy of visfatin in distinguishing obese T2DM patients from healthy controls. According to the results, visfatin exhibited low diagnostic accuracy, with an AUC < 0.70 (Figure 1). AUC values were interpreted as follows: AUC < 0.70 indicates low diagnostic accuracy; 0.70–0.90 indicates moderate diagnostic accuracy; and AUC > 0.90 indicates high diagnostic accuracy [26].

### 3.2. Biochemical Parameters

The biochemical parameters of the groups included in the study are summarised in Table 2. HbA1c and fasting plasma glucose levels were found to be significantly higher in the diabetic groups compared to the control group (*p* < 0.001 for both). No significant difference was observed in HbA1c and glucose levels between the obese and non-obese T2DM groups (Table 2). Insulin and HOMA-IR values were not calculated for the control group as insulin was not measured. In the obese T2DM group, insulin levels (*p* = 0.007) and HOMA-IR (*p* = 0.029) were significantly higher compared to the non-obese T2DM group. These findings support the effect of obesity in increasing insulin resistance (Table 3).

## 4. Discussion

In this study, we examined the serum levels of visfatin and chemerin—two adipokines involved in metabolic and inflammatory pathways—in obese and non-obese patients with T2DM. Our findings revealed that visfatin levels were significantly elevated in obese T2DM patients compared to healthy controls, whereas chemerin levels did not significantly differ among the study groups.

Obesity, particularly visceral obesity, has been identified as a significant contributing factor to the onset of insulin resistance and T2DM. The presence of chronic inflammation, which is a hallmark of obesity, has been demonstrated to play a pivotal role in the development of insulin resistance and the emergence of other obesity-related comorbidities [27]. Adipokines are biomolecules that play an important role in the pathophysiology of metabolic diseases such as obesity and T2DM [28]. The focus of this study is visfatin and chemerin, which have been demonstrated to be implicated in lipid and glucose metabolism, as well as inflammatory processes. Earlier research has indicated that other adipokines, including adiponectin, can serve as biomarkers for metabolic processes in prediabetes, T2DM and obesity [29,30,31,32].

Visfatin, also known as NAMPT, has been previously associated with insulin resistance, visceral adiposity, and systemic inflammation. Consequently, it shows promise as a biomarker in the diagnosis and monitoring of insulin resistance, T2DM, gestational diabetes and related complications [28]. The present study revealed that visfatin levels were significantly higher in obese T2DM patients compared to the control group. This finding suggests a potential role for visfatin in metabolic processes associated with obesity and T2DM. Visfatin, which regulates glucose homeostasis through nicotinamide adenine dinucleotide biosynthesis and exhibits insulin-like effects, may be considered a response to regulate blood glucose levels with its increased levels in obesity. However, it has been suggested that high visfatin levels contribute to the development of inflammation, insulin resistance, T2DM, as well as cardiovascular and renal diseases [28].

However, in our study, effect size analyses revealed very small differences in visfatin levels between groups (Cohen’s d < 0.2), and ROC analysis demonstrated poor diagnostic accuracy (AUC = 0.661, 95% CI: 0.538–0.784). These findings indicate that visfatin alone has limited clinical value and may only be informative when evaluated as part of multi-marker panels rather than as a standalone biomarker. For instance, Alnowihi et al. reported that serum visfatin levels were significantly higher in obese women compared to lean and overweight groups, and that these levels were positively correlated with BMI, waist circumference, hip circumference, insulin and HOMA-IR [33]. Similarly, Hetta et al. reported that serum visfatin levels were significantly higher in T2DM patients compared to healthy subjects and that these levels were positively correlated with BMI, waist circumference, HOMA-IR and proinflammatory markers. These findings emphasize the role of visfatin in insulin resistance pathogenesis and its relationship with proinflammatory cytokine activation [34]. In addition, many studies in the literature have reported a positive correlation between visfatin levels and BMI [23,24,25], but several other studies have not confirmed this association [3,35]. Despite the statistical significance, the ROC analysis showed that visfatin had limited diagnostic utility in distinguishing obese T2DM patients from healthy individuals, with an AUC value below 0.70. This suggests that while visfatin may be mechanistically relevant, it may not serve as a robust stand-alone biomarker for clinical use. Nevertheless, it may still have value in multi-marker panels or in tracking disease progression and treatment response. Further studies with larger samples and in different populations will contribute to a better understanding of the mechanisms affecting visfatin levels.

Chemerin is an adipokine produced by white adipose tissue and other tissues and plays an active role in the pathogenesis of inflammatory and metabolic diseases in various organs [36]. Recent studies have demonstrated the association of chemerin with glycemic control disorders, insulin resistance, T2DM and gestational diabetes [32,37,38,39]. Interestingly, we did not observe a significant difference in serum chemerin levels between the groups. This finding contrasts with several studies that reported elevated chemerin levels in T2DM and obesity. One explanation may be that chemerin expression is influenced not only by adiposity but also by proteolytic processing, which can be altered in different metabolic states. Studies investigating the association of chemerin with T2DM and obesity are available in the literature, but joint studies evaluating these two diseases at the same time are limited. Previous studies have reported both increased and unchanged chemerin levels in T2DM and obesity. Such inconsistencies may result from differences in sample size, age mismatch between study groups, or unmeasured confounders such as lifestyle and inflammatory factors. In our study, the absence of significant differences, together with negligible effect sizes, reinforces the complexity of chemerin regulation and suggests that multiple overlapping mechanisms, including proteolytic processing, may be involved. This interpretation aligns with previous reports indicating that chemerin activity, rather than total concentration, may better reflect metabolic dysregulation in T2DM.

In most studies, chemerin levels were positively correlated with markers of poor glycemic control, fasting plasma glucose, fasting insulin, HbA1c and HOMA-IR [36,40,41,42,43]. It is interesting to note that these correlations remained consistent following adjustment for factors such as age, gender and BMI [39,44]. It has been hypothesised that chemerin exerts its effects on insulin signalling pathways, potentially contributing to insulin resistance through the process of inducing inflammation [36,45]. In particular, it is hypothesised that chemerin interferes with this process by disrupting the functioning of certain proteins in the insulin signalling chain [36,45]. A number of studies have demonstrated that alterations to one’s lifestyle have the potential to decrease chemerin levels and enhance insulin resistance [46]. Contrary to the findings reported in the extant literature, a study planned with a similar design examined the differences in the concentrations of various forms of chemokine in individuals with T2DM but with different BMI. The study found that total chemerin levels were similar across all groups, which corroborates the findings of the present study. However, it was stated that obesity status affected the proteolytic processing of chemerin, suggesting that T2DM may equalize chemerin levels [47]. Zhang et al. discovered a positive correlation between chemerin and IL-6 levels, thereby suggesting that chemerin may play a significant role in obesity-related inflammation [48]. It is hypothesised that chemerin plays a pivotal role in various disease processes, including inflammation, T2DM and metabolic syndrome. The potential involvement of chemerin in the dysregulation of these processes is a subject of ongoing research. In addition, the mediating role of chemerin in the development of obesity and T2DM suggests that this adipokine may offer a potential approach to diagnosis and treatment as a biomarker in the future [17,49]. The novelty of our study lies not in identifying new biomarkers but in confirming the limited diagnostic accuracy of visfatin and highlighting the complex regulation of chemerin, thus providing additional evidence to the current literature.

This study has certain limitations. Its cross-sectional design precludes causal inferences, and the relatively small sample size, particularly in the control group, may limit the generalizability of the results. The gender distribution is not homogeneous across the study groups, and this may have a partial effect on metabolic parameters. Insulin measurements were not obtained in the control group for ethical and financial reasons, which prevented direct comparison of HOMA-IR across all groups. Anthropometric measurements were obtained using a body composition analyser Tanita MC-780MA, Tokyo, Japan) to ensure standardized BMI calculation; however, additional body composition data such as fat mass or visceral fat were not systematically recorded and thus excluded from the analysis. Furthermore, lifestyle and inflammatory factors were not fully controlled, which may have influenced adipokine levels. Despite these limitations, the study provides useful insights into the relationship between adipokines, obesity, and metabolic regulation in T2DM. Another limitation of our study is that the gender distribution was not homogeneous among the study groups, which might have a partial influence on metabolic parameters. However, this imbalance reflects the real-world population characteristics of patients with type 2 diabetes.

## 5. Conclusions

Our study demonstrated that serum visfatin levels were significantly higher in obese patients with T2DM than in healthy controls. Although visfatin levels were statistically higher, effect size analyses revealed very small differences and ROC analysis showed poor diagnostic accuracy (AUC < 0.70). Therefore, visfatin alone has limited clinical utility and may only be relevant as part of multi-marker panels. In contrast, no significant difference in serum chemerin levels was observed among the study groups. This finding highlights the complexity of chemerin regulation and indicates that its function may be affected by factors other than obesity or diabetes. Further large-scale, longitudinal studies are needed to clarify the temporal and mechanistic links between these adipokines and metabolic disease progression.

## Figures and Tables

**Table 1:** Baseline clinical and demographic features of the patients and controls.

Characteristic	Controls (*n*: 36)	Non-Obese T2DM (*n*: 60)	Obese T2DM (*n*: 74)	*p* Values
Gender, F/M, (*n*/%)	22/14 (61.1/38.9)	19/41 (31.7/68.3)	47/27 (63.5/36.5)	<0.001
Age (years)	44.47 ± 8.00	55.56 ± 9.67	54.02 ± 6.78	0.07
Visfatin (ng/mL)	30.25 ± 26.40	34.37 ± 26.48	33.00 ± 20.61 *	0.01
Chemerin (ng/mL)	728.02 ± 557.95	690.09 ± 639.83	644.66 ± 591.01	0.42
Height (cm)	166.41 ± 10.58	164.93 ± 8.69	158.09 ± 9.31	0.13
Weight (kg)	68.83 ± 20.48	73.10 ± 11.20	89.71 ± 14.63 *^#^	<0.001
BMI (kg/m^2^)	24.80 ± 6.79	26.80 ± 2.80	35.96 ± 5.69 *^#^	<0.001
Waist Circumference (cm)	92.14 ± 10.36	93.93 ± 9.28	105.60 ± 9.44 *^#^	<0.001
Smoking, Yes/No, (*n*/%)	8/28 (22.2/77.8)	9/51 (15.0/85.0)	12/62 (16.2/83.8)	
Family History with DM, Yes/No, (*n*/%)	16/10 (52.77/27.77)	48/12 (76.66/16.66)	62/12 (78.37/13.51)	

Clinical and demographic characteristics of the study groups. Values are presented as mean ± SD or *n* (%). For continuous variables, the Kruskal–Wallis test was used to compare the three groups. When significant differences were detected, pairwise comparisons were performed using the Bonferroni-corrected Mann–Whitney U test (adjusted *p* < 0.017). Categorical variables were analyzed using the chi-square test. * Levels were significantly higher in obese T2DM patients compared to the control group. ^#^ Levels were significantly higher in obese T2DM patients compared to the non-obese T2DM patients F: Female, M: Male, T2DM: Type 2 Diabetes Mellitus, BMI: Body Mass Index.

**Table 2:** HbA1c and Glucose Levels of Study Groups.

Biochemical Parameters	Controls (*n*: 36)	Non- Obese T2DM (*n*: 60)	Obese T2DM (*n*: 74)	*p* Values
HbA1c (%)	5.5 ± 1.0	8.4 ± 1.9 *	7.6 ± 2.3 *	<0.001
Glucose (mg/dL)	85.0 ± 15.7	192.1 ± 65.7 *	174.8 ± 66.2 *	<0.001

HbA1c and Glucose levels of the study groups. Values are presented as mean ± SD. * *p* < 0.05 vs. Control group (Bonferroni-adjusted pairwise comparisons).

**Table 3:** Insulin and HOMA-IR Levels of Study Groups.

Biochemical Parameters	Non- Obese T2DM (*n*: 60)	Obese T2DM (*n*: 74)	*p* Values
Insulin (µIU/mL)	8.8 ± 8.9	12.3 ± 11.6 †	0.007
HOMA-IR	4.1 ± 4.2	5.1 ± 5.6 †	0.029

Insulin and HOMA-IR levels of the study groups. Values are presented as mean ± SD. † *p* < 0.05 between T2DM subgroups (Obese T2DM vs. Non-obese T2DM). Insulin and HOMA-IR values were not measured in the Control group; therefore, comparisons were performed only between T2DM subgroups.

## Data Availability

Data supporting the findings of this study are available from the corresponding author upon reasonable request.

## Abbreviations

ADA	American Diabetes Association
AUC	Area under the curve
BMI	Body mass index
ELISA	Enzyme-linked immunosorbent assay
HbA1c	Glycated hemoglobin
HOMA-IR	Homeostatic Model Assessment of Insulin Resistance
NAMPT	Nicotinamide phosphoribosyl transferase
PBEF	pre-B cell colony enhancer factor
ROC	Receiver operating characteristic
T2DM	Type 2 diabetes mellitus
vWAT	Visceral white adipose tissue
